# Does dynamic balance affect cube mental rotation task in badminton vs. volleyball female players?

**DOI:** 10.1186/s40359-024-01589-w

**Published:** 2024-03-07

**Authors:** Samiha Amara, Badriya Al-Hadabi, Heba El-Ashkar, Nabil Gmada, Hamdi Habacha, Bessem Mkaouer

**Affiliations:** 1 Physical Education and Sport Sciences Department, College of Education, Sultan Qaboos University, Muscat, Sultanate of Oman; 2 Higher Institute of Sport and Physical Education of Ksar Said, Manouba University, Tunis, Tunisia

**Keywords:** Mental rotation, Response time, Dynamic balance, Badminton, Volleyball

## Abstract

**Background:**

Changing from a static to a dynamic balance condition could affect the performance of a cognitive task such as mental rotation. Thus, the main goal of this study is to investigate aspects of visual-spatial cognition between two non-contact sports (i.e., badminton and volleyball) in different upright conditions (i.e., standing position, frontal balance, and sagittal balance).

**Methods:**

Thirty-five volunteer female sports and physical education students, fourteen specialists in badminton and twenty-one specialists in volleyball agreed to participate in this study. Each of the assessments was a 3D cube mental rotation task with and/or without balance exercises (i.e., frontal and/or sagittal balance) on a wobble board. Five stimuli were used in the mental rotation task (i.e., 45°, 135°, 180°, 225° and 315° for objected-based cube condition with egocentric transformation) which included pairs of standard and comparison images.

**Results:**

The findings indicate that there was a notable decrease (*p* < 0.001; *d* = 1.745) in response time in both dynamic balance conditions (i.e., frontal and sagittal balance) compared to standing position condition. In addition, results revealed significant interaction between balance conditions (i.e., frontal and/or sagittal balance) and groups (i.e., badminton and volleyball) in the response time at 225° angle and in the error percentage.

**Conclusions:**

In sum, dynamic balance is also an activity that involves mental manipulation of objects in 3D space, which can enhance badminton and volleyball female players’ ability to rotate 3D cube stimuli.

## Background

Mental rotation (MR) is the ability to rotate mental representations of objects in one’s mind. This ability is thought to be important for tasks such as visual search, object recognition, and spatial reasoning. Steggemann et al. [1] have shown that MR ability is related to the way in which the brain processes visual information. Therefore, Shepard, Metzler [2] introduced the concept of MR, defining it as the process of imagining an object rotating away from its original position. In their research, the authors presented the participants with pairs of misoriented figures of asymmetric 3D cube assemblies and asked them to determine whether they depicted similar or mirror-reversed objects. The authors observed that the time required for judging (i.e., response time) increased as the linear function of angle rotation revealed correlation between real and imagined rotations [3].

Many studies have found that athletes who engage in sports activities, especially those that require spatial awareness and coordination, tend to have better mental rotational skills than non-athletes [4–8]. Furthermore, Jansen, Lehmann [9] showed that if motor skills are correlated with mental rotational performance, then arguably, individuals with advanced motor skills or high levels of physical activity should exhibit better mental rotational performance. In addition, it was found that cognitive skills and motor processes appear to play an important role in solving the MR task. Following this line of research, lots of studies consistently showed activation of motor cortical areas when performing a 3D MR task [10–12]. Then, we can conclude that activities require the use of spatial skills such as visualizing and manipulating objects in space to improve MR ability assessment. In this context, Jansen et al. [13] showed that 3 months of juggling training had a positive effect on a timed MR task using cubes compared to an untrained control group. Moreover, Moreau et al. [14] revealed that elite athletes who completed daily practice of a combat sport (i.e., fencing, judo, and wrestling) showed a higher MR performance than elite runners. Researchers have proven that athletes, such as judokas or wrestlers, who require linking visuospatial and kinaesthetic processes during their physical activity, show better mental rotational performance than athletes who primarily engage in cardiovascular activities such as running. Also, gymnasts who used mental and physical rotations in their practice showed a better MR performance than non-athletes [8]. Thus, athletes of different abilities in various sports appear to use different strategies to solve the same MR tasks, linking sensorimotor experiences to the perception of movement and form for these tasks [1, 15–17].

In this regard, one way to better understand the link between stability and cognitive processing is to investigate the effect of dynamic balance on cognitive performance. Particularly, balance exercises play a very important role in spatial orientation and visual perception [18] which is maintained by a complex set of sensorimotor control systems including sensory input from the visual, proprioceptive, and vestibular systems [19]. Bigelow, Agrawal [20] added that the cognitive areas of visuospatial ability, such as spatial memory, navigation, MR, and mental representation of three-dimensional space, were shown to be associated with vestibular function. According to Rogge et al. [21], balance training can improve memory and spatial cognition more than cardiorespiratory fitness. It’s possible that stimulating the vestibular system during balance training changes the hippocampus and parietal cortex, possibly through direct pathways between the vestibular system and these brain regions, thus improves abilities and MR tasks that appear to rely on bilateral parietal cortices and the hippocampus, respectively.

However, for team sports in this present study (such as badminton and volleyball, players), were often trained to perceive and analyze moving objects in relation to teammates and opponents. Thus, this requires a significant cognitive process, not only to understand one’s environment and navigate within it, but also to perform a wide range of cognitive tasks that require the visualization of possible situational transitions [22] which could enhance the ability to mentally rotate objects in a visual environment [1]. Consequently, the benefits of team sports may arise from the combination of visual and motor training in MR tasks. In addition, studies [23–25] have shown that athletes in team games and special racquet sports have significantly shorter response times than those in other sports. Also, Ozel et al. [8] and Wang et al. [26] studies found that badminton (BMT) players reacted faster to the MR task than non-badminton players. This is because BMT training exercises, require players to repeatedly exhibit the same response to an event. BMT players may thus gain the ability to quickly recall and encode information, resulting in shorter response times that can be transferred to visuospatial tests. On the other hand, BMT players confirmed their ability to respond faster to the 3D cube task, but their MR was no better than that of non-badminton players [27]. Furthermore, Delpont et al. [28] observed faster visual transmission in tennis and squash players compared to rowers and non-athlete controls. This benefit stems from the central nervous system effects of team games and specifically racquet sports, which require rapid visual activity and involve mental manipulation of objects in 3D space [10, 15]. Moreover, a highly specialized sport, volleyball (VB) is known for its quick pace, alternating high-intensity and low-intensity activity, and mix of offense and defense while standing in a low position to receive services. All these abilities boost spatial orientation, which in turn improves visual perception and MR [18].

In this context, few studies [29, 30] have investigated the effect of balance on the MR task or vice versa in sports science students, but no studies have compared MR performance between sport specialties like BMT and VB players, using 3D cube figures as stimulus material under various upright conditions (i.e., standing position, frontal balance, and sagittal balance). In this regard, the main goal of this study is to investigate aspects of visual-spatial cognition between two non-contact sports (i.e., BMT and VB) in different upright conditions (i.e., standing position, frontal balance, and sagittal balance). We hypothesized firstly, that the dynamic balance conditions have immediate beneficial effects on MR tasks by decreasing response time for both BMT and VB players. Secondly, players engaged in BMT are expected to demonstrate superior ability and faster response times compared to VB players in recognizing the correct orientation of rotated 3D cube images [29, 30].

## Methods

### Participants

A minimum sample size of 35 participants (i.e., 14 badminton and 21 volleyball female players) was determined from an a priori statistical power analysis using G*Power software (Version 3.1, University of Dusseldorf, Germany [31]). The power analysis was computed with an assumed power at 0.95 at an alpha level of 0.05 and a moderate effect size (*f* = 0.30, *d* = 0.60 and critical F = 3.150). Therefore, thirty-five volunteer female sports and physical education students, fourteen specialists in BMT (age 20.48 ± 1.04 years; height 1.80 ± 0.03 m; weight 78.12 ± 3.73 kg; experience 6 ± 2 years; average training 8 ± 2 h/week) and twenty-one specialists in VB (age 21.57 ± 1.47 years; height 1.87 ± 0.02 m; weight 80.03 ± 4.03 kg; experience 6 ± 2 years; average training 8 ± 2 h/week) played at national level, agreed to participate in this study. After being informed in advance of the procedures, methods, benefits, and possible risks of the study, each participant reviewed and signed a consent form to participate in the study. The experimental protocol was performed in accordance with the Declaration of Helsinki for human experimentation [32] and was approved by the University Local Ethical Committee (EDU/PHEDS83961/2022).

### Experimental design and procedures

This study is made up of three random assessments (i.e., randomized counterbalanced, Latin Square [33]). Every assessment took place on a separate day successively. All assessments were carried out in the gymnasium at the same time of the day (i.e., between 10:00 PM and 12:00 PM). Each of the assessments was a cube MR task with and/or without balance exercises (i.e., frontal and/or sagittal balance) on a wobble board (i.e., single plane balance board (SPBB) length and width 420 × 420 mm; height 70 mm [34–37]).

Each participant stands at 1 m distance facing the screen, either in static and/or dynamic balance (i.e., frontal and/or sagittal balance) conditions, on SPBB, with a wireless joystick (Bluetooth) in their hand. The objective is to respond as quickly as possible to stimuli (3D rotated cube) within a maximum allowed duration of 4 min to end experimentation.

**Fig. 1:**
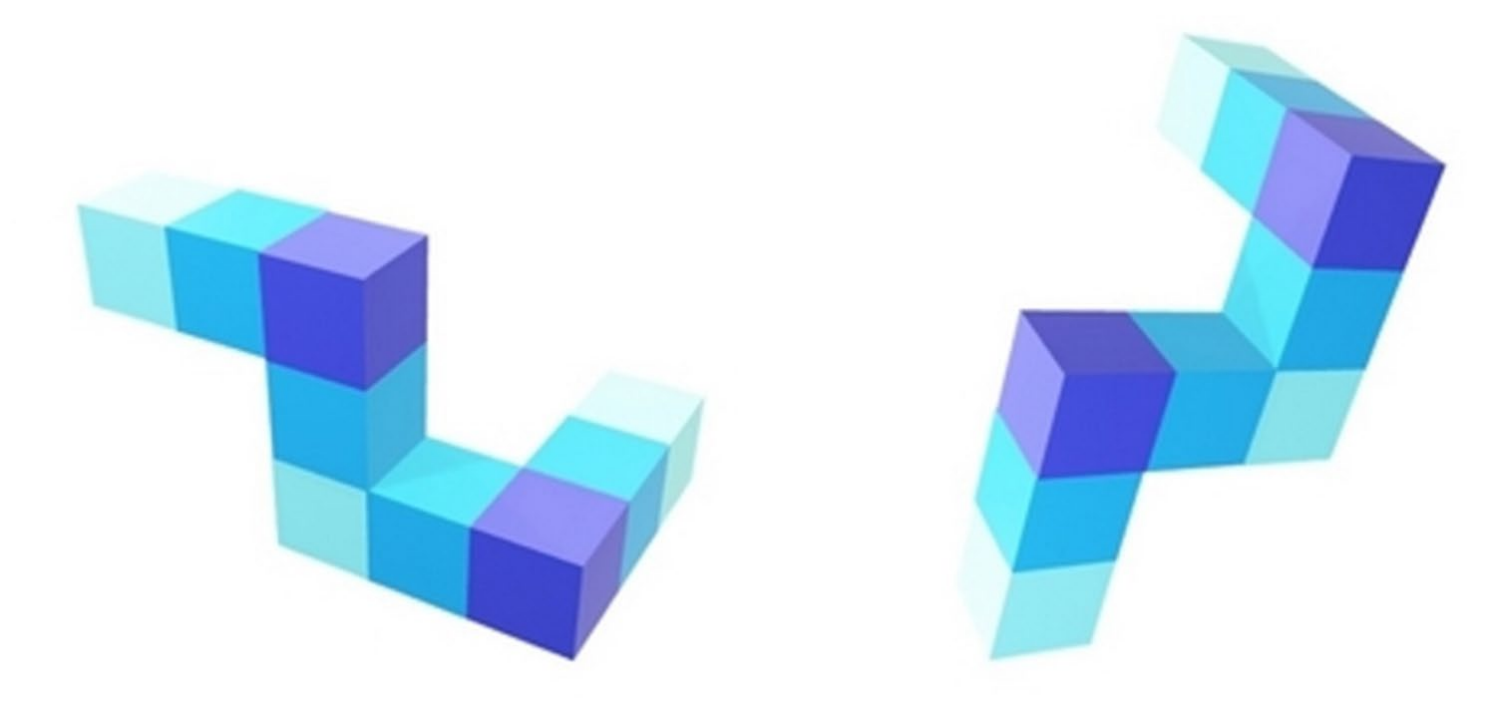
Example of stimulus objected-based cube conditions

Five stimuli were used in the MR task (i.e., 45°, 135°, 180°, 225° and 315° for objected-based cube condition) including pairs of standard and comparison images (Fig. 1). We used the standard image on the left part of the monitor screen and the rotated one on the right of the screen. The comparison image was rotated in one of five orientations (i.e., 45°, 135°, 180°, 225°, and 315°) and displayed at the right of the screen [1, 38–41].

**Fig. 2:**
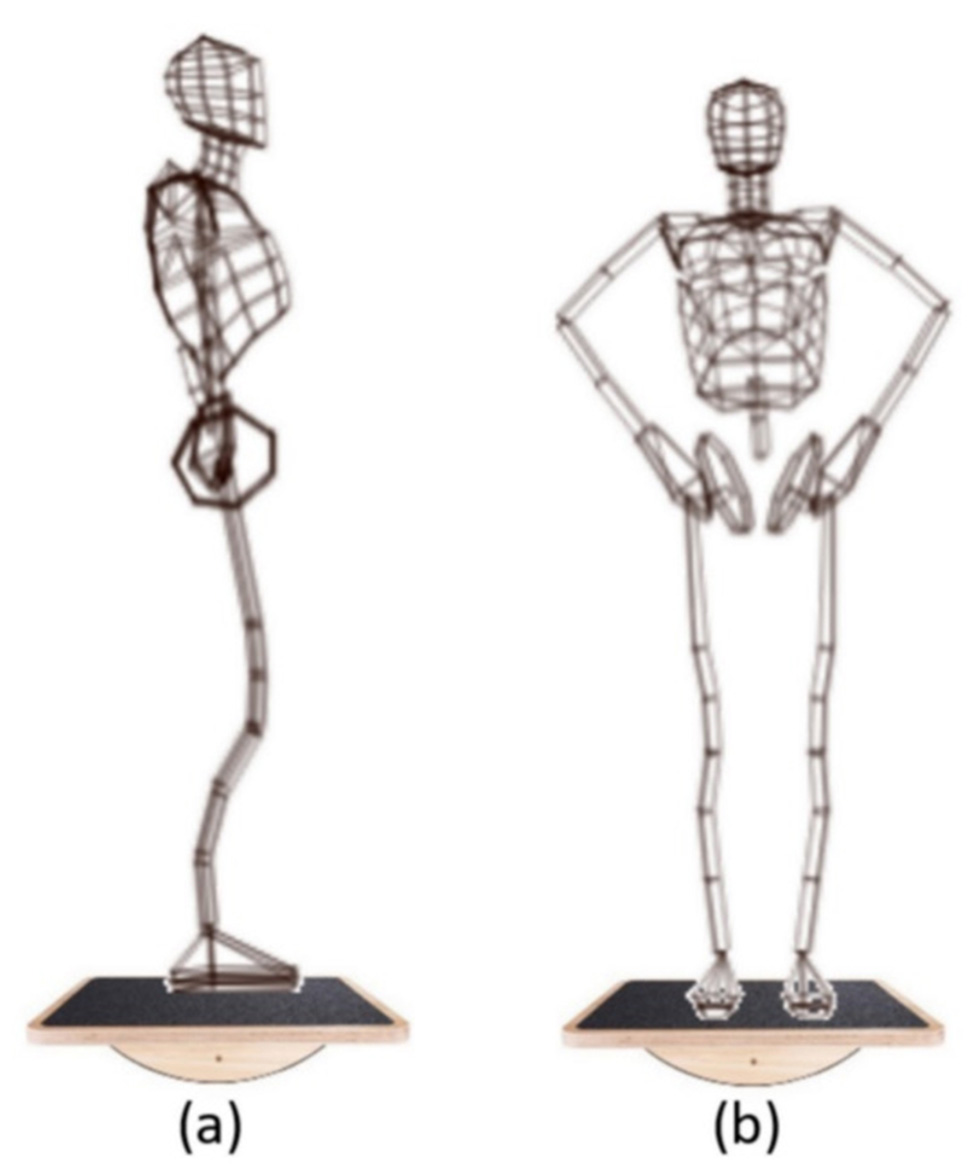
Experimental protocol: (**a**) Bipedal sway, sagittal balance; (**b**) Bipedal sway, frontal balance [35]

The MR task will be studied in three conditions, with each condition on a separate day and an interval of 24 h:

- In standing position (SP): Participant takes an upright position in front of the PC with a wireless joystick in her hand. The subject was asked to respond as precisely and quickly as possible to the displayed stimuli/figures (i.e., pairs of 3D rotated cubes) by indicating correctness with the left button for correct responses (i.e., same figures) and the right button for incorrect responses (i.e., different figures).
- In sagittal balance (SB): The subject takes an upright position on the SPBB (i.e., placed on the z-axis for anteroposterior sway) in front of the PC with a wireless joystick in her hand. Subject was asked to respond as precisely and quickly as possible to the displayed stimuli/figures (i.e., pairs of 3D rotated cubes) by indicating correctness with the left button for correct responses (i.e., same figures) and the right button for incorrect responses (i.e., different figures), (Fig. 2a).

(c) In frontal balance (FB): The subject takes an upright position on the SPBB (i.e., placed on the x-axis for mediolateral sway) in front of the PC with a wireless joystick in her hand. Subject was asked to respond as precisely and quickly as possible to the displayed stimuli/figures (i.e., pairs of 3D rotated cubes) by indicating correctness with the left button for correct responses (i.e., same figures) and the right button for incorrect responses (i.e., different figures), (Fig. 2b).

This results in 3 conditions (i.e., static, sagittal and frontal balance) × 2 groups (i.e., BMT and VB) × 5 angle display (i.e., 45°, 135°, 180°, 225°, and 315°) × 2 response possibilities (i.e., left-right or same-different) trials for a total of 60 trials. The order of stimuli presentation will be counterbalanced, and each rotation angle could not appear 2-times successively.

Each trial begins with a blank screen for 1000ms, after which a black fixation cross was displayed at the center for 500ms. After fixation, the test image is presented for a maximum of 5000ms and remains on the screen until a response was given. Stimuli will be display and response times (RT) and error percentage (EP) will be recorded via the free software OpenSesame [42]. The MR task lasts about 4 min.

Two AEE PNJ cameras (SD18, HD 720p, CCD 1,000,000 pixels, SSC 1/4000 per second, minimum sensitivity 1 lx, acquisition frequency 120 Hz, zoom angle 145°) were used to perform a two-dimensional (2D) kinematic balancing study. The first camera was placed 2 m in front of the single-plane balance board (SPBB), while the second camera was positioned 2 m to the side of the SPBB to record sway movement. Twenty reflective markers were affixed to every participant using the Hanavan model [43] modified by de Leva [44] digitized through the video-based data analysis system SkillSpector® (Version 1.3.2, Odense SØ– Denmark [45]) with quantic-spline data filtering. The sway velocity and displacement of the center of mass (COM) were studied for both dynamic balance conditions (i.e., FB and SB) on the SPBB.

### Statistical analysis

The data analysis was done using the SPSS 20 package (SPSS, Chicago, IL, USA) software as part of the statistical analysis. Data are reported as mean ± standard deviation (SD) and 95% confidence intervals (95% CI). Effect size (*d*) was calculated using G*Power software [Version 3.1, University of Dusseldorf, Germany]. The following scale was used for the interpretation of d: < 0.2 (trivial); 0.2–0.6 (small); 0.6–1.2 (moderate); 1.2–2.0 (large); and > 2.0 (very large) [46]. The normality of distribution estimated by the Kolmogorov-Smirnov test was acceptable for all variables (*p* > 0.05). Therefore, repeated measures ANOVA were applied to compare the different balance conditions (i.e., standing position, sagittal balance, and frontal balance) and groups (i.e., BMT and VB). Pairwise comparison was conducted using Bonferroni post-hoc test. Additionally, effect sizes (*d*) were determined from ANOVA output by converting partial eta-squared to Cohen’s *d*. The level of significance was set at *p* < 0.05.

## Results

The repeated measures ANOVA showed a significant difference between conditions (i.e., SP, FB, and SB), *p* < 0.01), and groups (i.e., BMT and VB) in the RT with (*p* < 0.01). In addition, results revealed significant (*p* < 0.05) interaction between balance conditions (i.e., SB and FB) and groups (i.e., BMT and VB) in the RT at 225° angle and in the general error percentage (EP) (Table 1).

**Table 1:** ANOVA with repeated measures

Source		df	Mean Square	F	Sig.	Effect Size	Power
Balance	RT Gen	2	11025770.803	25.423	0.000**	1.745	1.000
	EP Gen	2	54.678	1.090	0.342	0.363	0.233
	RT 45°	2	6453114.037	20.304	0.000**	1.569	1.000
	EP 45°	2	93.004	0.532	0.590	0.255	0.134
	RT 90°	2	5188452.130	11.545	0.000**	1.182	0.992
	EP 90°	2	98.612	0.431	0.652	0.229	0.117
	RT 135°	2	23045872.614	27.506	0.000**	1.827	1.000
	EP 135°	2	384.923	1.741	0.183	0.458	0.353
	RT 180°	2	10786066.101	9.649	0.000**	1.080	0.977
	EP 180°	2	495.673	1.657	0.198	0.449	0.338
	RT 225°	2	14572770.848	12.891	0.000**	1.250	0.996
	EP 225°	2	472.017	2.108	0.130	0.505	0.418
	RT 270°	2	11193959.412	13.520	0.000**	1.281	0.997
	EP 270°	2	83.336	0.296	0.745	0.190	0.095
	RT 315°	2	10018628.416	16.705	0.000**	1.422	1.000
	EP 315°	2	3.080	0.021	0.979	0.063	0.053
Sports	RT Gen	1	2436765.897	3.095	0.088	0.613	0.401
	EP Gen	1	2086.921	2.596	0.117	0.561	0.346
	RT 45°	1	2952768.799	5.321	0.027*	0.803	0.610
	EP 45°	1	1483.071	2.608	0.116	0.561	0.348
	RT 90°	1	4668474.777	5.966	0.020*	0.850	0.659
	EP 90°	1	6216.824	5.716	0.023*	0.833	0.641
	RT 135°	1	1110791.703	0.805	0.376	0.313	0.141
	EP 135°	1	670.552	0.597	0.445	0.270	0.117
	RT 180°	1	68875.972	0.027	0.871	0.063	0.053
	EP 180°	1	1304.727	0.879	0.355	0.326	0.149
	RT 225°	1	2099391.868	1.059	0.311	0.357	0.170
	EP 225°	1	4303.951	3.005	0.092	0.601	0.391
	RT 270°	1	1647875.023	1.188	0.284	0.308	0.185
	EP 270°	1	3571.333	2.674	0.112	0.569	0.355
	RT 315°	1	3784070.801	3.606	0.066	0.663	0.454
	EP 315°	1	1238.639	1.275	0.267	0.392	0.195
Balance * Sports	RT Gen	2	867129.718	1.999	0.144	0.491	0.399
	EP Gen	2	164.386	3.276	0.044*	0.629	0.604
	RT 45°	2	659783.928	2.076	0.134	0.500	0.412
	EP 45°	2	384.053	2.197	0.119	0.514	0.433
	RT 90°	2	1278716.915	2.845	0.065	0.585	0.540
	EP 90°	2	66.370	0.290	0.749	0.190	0.094
	RT 135°	2	276898.445	0.330	0.720	0.201	0.101
	EP 135°	2	83.307	0.377	0.687	0.210	0.108
	RT 180°	2	402276.416	0.360	0.699	0.210	0.105
	EP 180°	2	755.879	2.527	0.088	0.552	0.489
	RT 225°	2	3825059.929	3.384	0.040*	0.640	0.618
	EP 225°	2	227.544	1.016	0.368	0.351	0.220
	RT 270°	2	1296550.151	1.566	0.217	0.434	0.321
	EP 270°	2	194.421	0.690	0.505	0.285	0.162
	RT 315°	2	449979.784	0.750	0.476	0.300	0.172
	EP 315°	2	399.953	2.725	0.073	0.573	0.521

(RT) Response time; (EP) Error percentage; (Gen) General; (*) Significant at *p* < 0.05; (**) Significant at *p* < 0.001

**Fig. 3:**
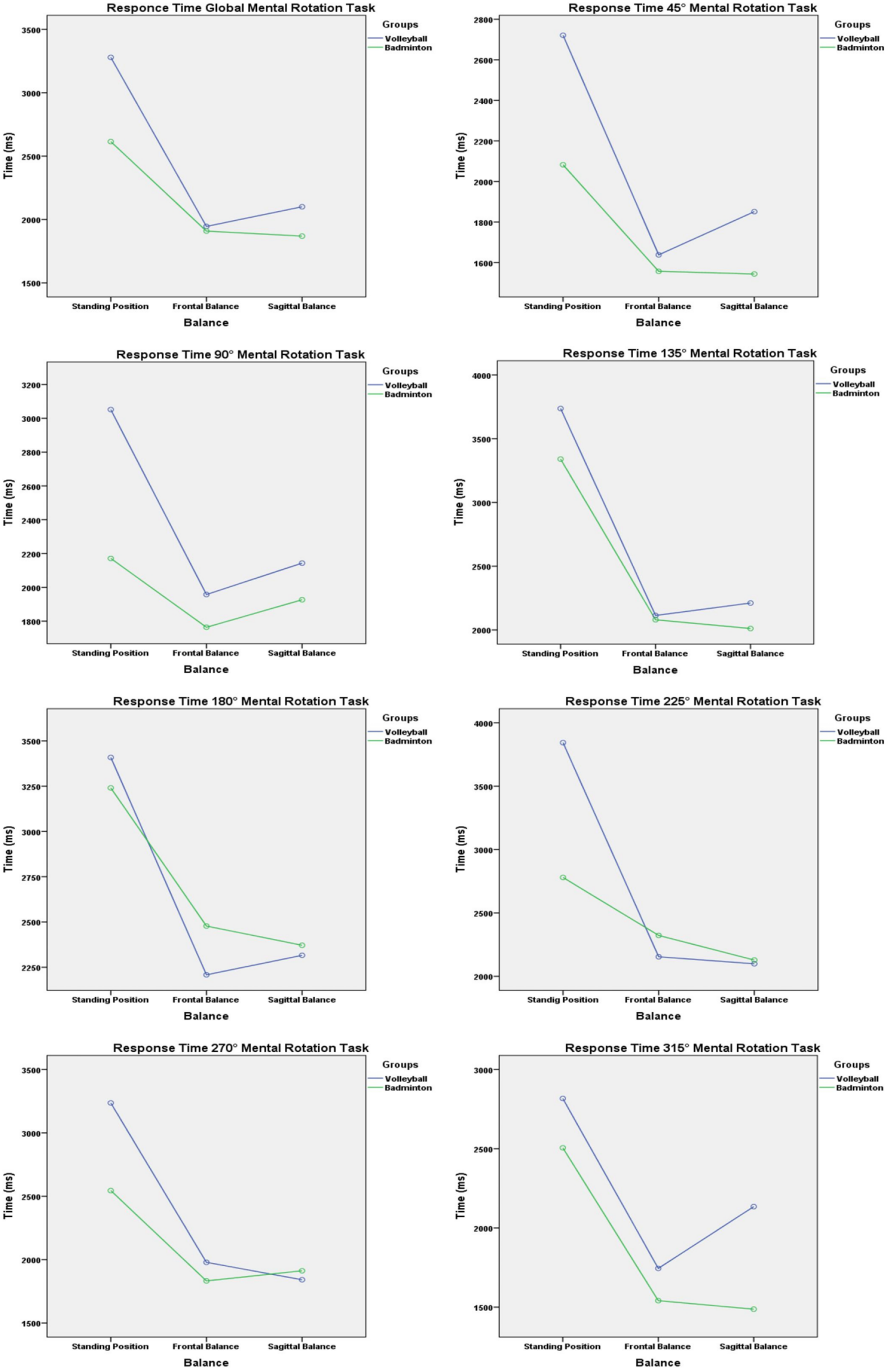
Response time of groups vs. balance conditions in different mental rotation angles

Pairwise comparison between MR conditions (i.e., SP, SB, and FB) showed significant difference (*p* < 0.01) for RT in all rotation degrees (i.e., 45°, 90°, 135°, 180°, 225°, 270°, and 315°) between SP vs. FB and between SP vs. SB conditions (Table 2; Fig. 3).

**Table 2:** Pairwise comparison

Measure		Mean Diff	Std. Error	Sig.	Effect Size
RT Gen	SP vs. FB	1019.760	178.332	0.000**	5.728
	SP vs. SB	962.108	179.749	0.000**	5.374
	FB vs. SB	57.652	115.476	0.621	0.495
RT 45°	SP vs. FB	804.073	155.891	0.000**	5.187
	SP vs. SB	704.147	151.127	0.000**	4.663
	FB vs. SB	99.927	98.043	0.316	1.010
RT 90°	SP vs. FB	750.811	158.681	0.000**	4.751
	SP vs. SB	576.568	189.796	0.005**	3.050
	FB vs. SB	174.243	138.019	0.216	1.260
RT 135°	SP vs. FB	1441.656	267.137	0.000**	5.399
	SP vs. SB	1427.148	233.007	0.000**	6.125
	FB vs. SB	14.508	154.791	0.926	0.090
RT 180°	SP vs. FB	981.735	264.738	0.001**	3.718
	SP vs. SB	980.958	286.039	0.002**	3.429
	FB vs. SB	0.777	218.420	0.997	0.001
RT 225°	SP vs. FB	1073.192	284.994	0.001**	3.778
	SP vs. SB	1197.909	274.484	0.000**	4.371
	FB vs. SB	124.716	212.842	0.562	0.584
RT 270°	SP vs. FB	985.027	242.755	0.000**	4.070
	SP vs. SB	1013.812	244.763	0.000**	4.154
	FB vs. SB	28.784	170.324	0.867	0.164
RT 315°	SP vs. FB	1018.559	209.184	0.000**	4.873
	SP vs. SB	850.489	208.869	0.000**	4.088
	FB vs. SB	168.070	140.400	0.240	1.200

(RT) Response time; (EP) Error percentage; (Gen) General; (SP) Standing position; (FB) Frontal balance; (SB) Sagittal balance; (*) Significant at *p* < 0.05; (**) Significant at *p* < 0.001

Furthermore, between group comparison (i.e., BMT vs., VB), showed a significant difference (*p* < 0.05) in the RT at 45 degrees (2720.83 ± 800.45ms vs. 2082.08 ± 898.08ms, respectively VB and BMT players) and 90 degrees (3051.77 ± 846.18ms vs. 2171.02 ± 932.79ms, respectively VB and BMT players) and the EP at 90 degrees (26.60 ± 23.34 vs. 10.71 ± 20.26, respectively VB and BMT players).

In addition, the interaction between balance * groups showed a significant difference in the global EP (22.35 ± 18.19, respectively) and in RT only at 225° angle in the SP vs. FB and SP vs. SB conditions (3417.701597.64ms vs. FB = 2221.281085.11ms vs. SB = 2110.50822.17ms, respectively VB and BMT players) (*p* < 0.05) between BMT and VB players.

**Fig. 4:**
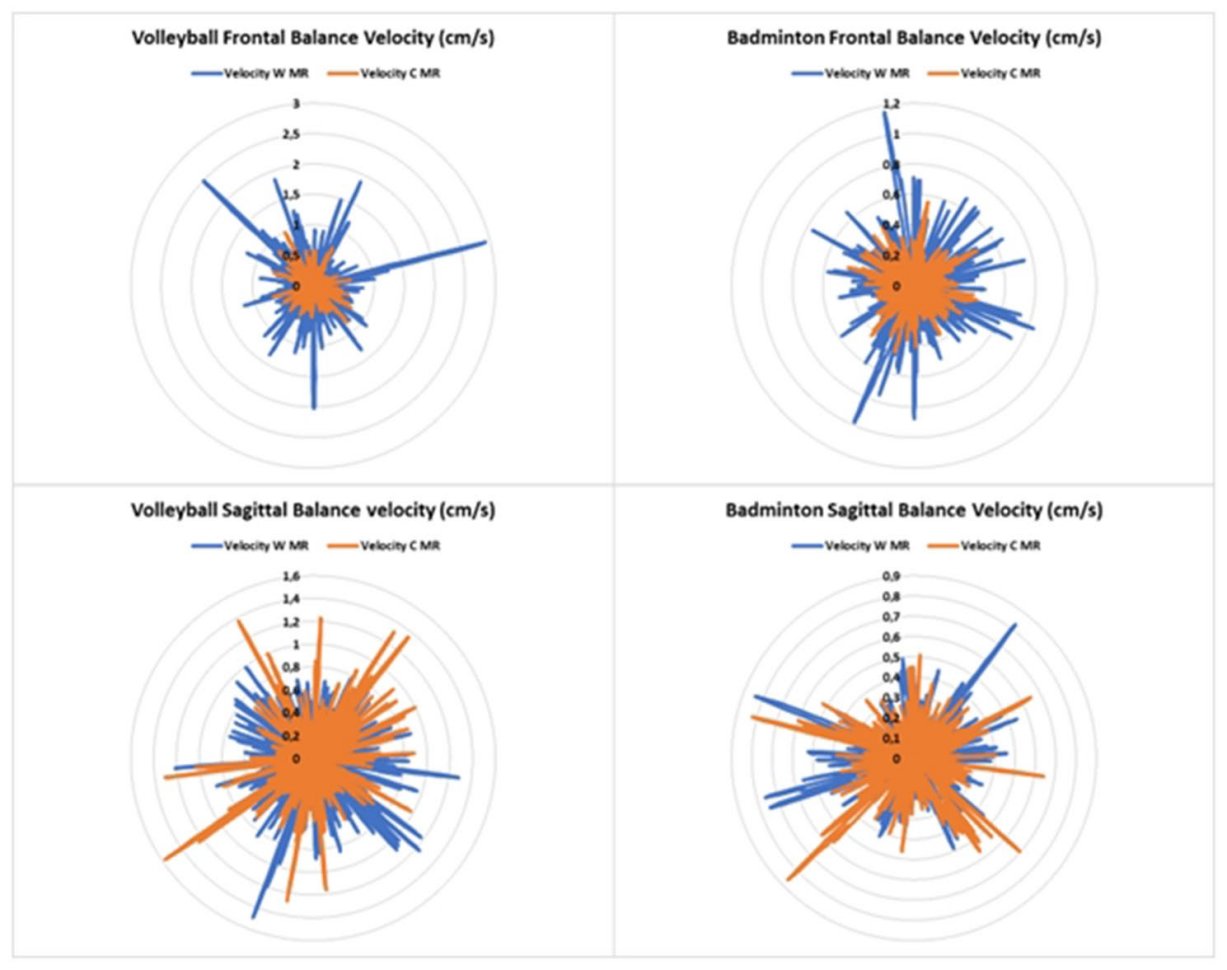
Sway velocity in frontal and sagittal conditions with and without cube mental rotation task

**Fig. 5:**
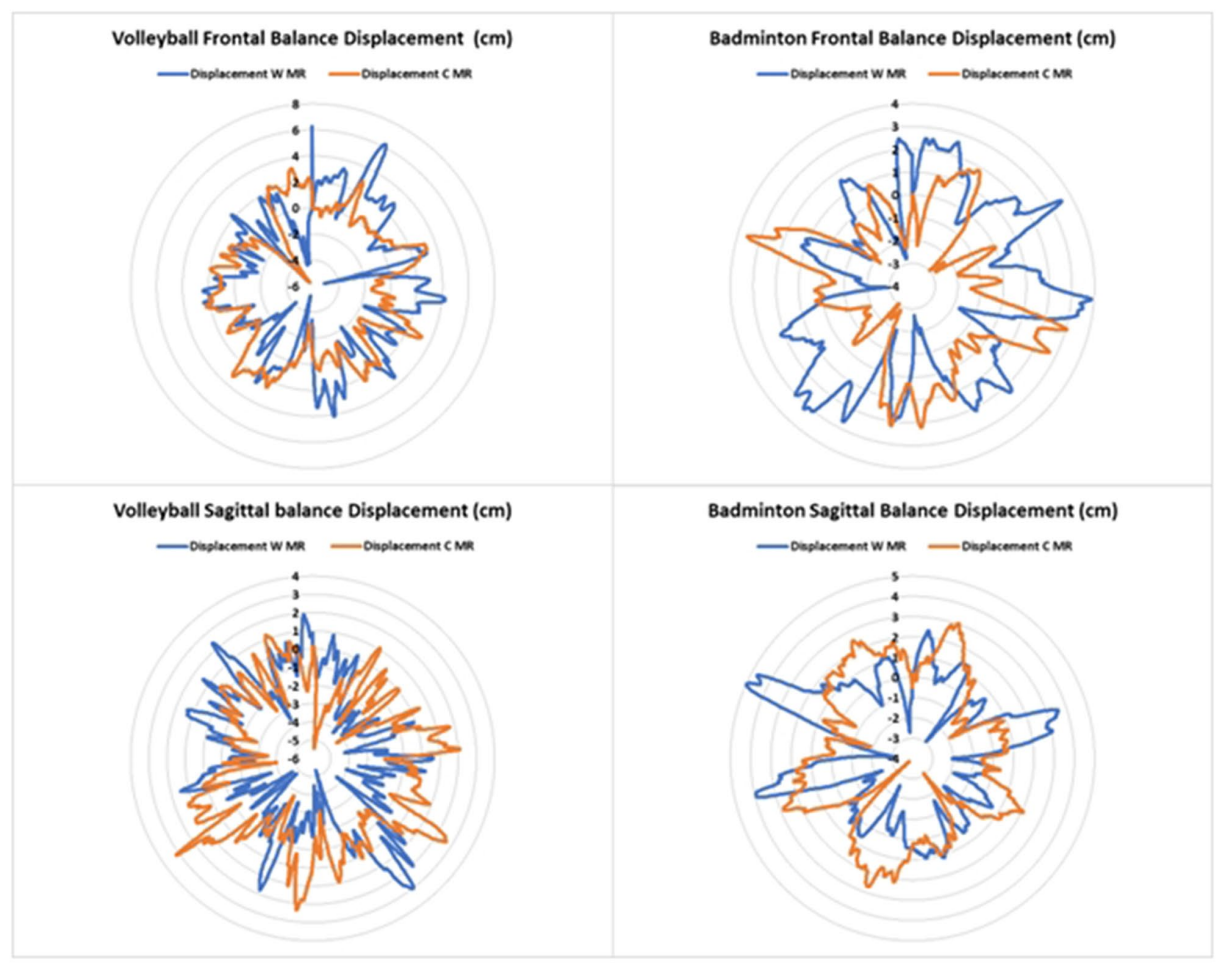
Sway displacement in frontal and sagittal conditions with and without cube mental rotation task

In the other side, balance (i.e., velocity and displacement) was enhanced when introducing MR task (*p* < 0.01) in both sport disciplines (i.e., volleyball and badminton) and balance conditions (i.e., FB and SB) (Figs. 4 and 5). In addition, there is a significant difference between sports in the displacement (8.866 ± 2.851 cm vs. 7.609 ± 2.698 cm; F_(1,33)_ = 11.151; *p* < 0.01; *d* = 1.632, respectively VB and BMT players) and a significant interaction FB * sports in the sway velocity (3.234 ± 1.640 cm/s vs. 2.471 ± 0.439 cm/s; F_(1,33)_ = 10.667; *p* < 0.001; *d* = 1.362, respectively VB and BMT players) in favor of badminton players.

## Discussion

The purpose of this study was to compare the MR performance in different upright conditions (i.e., SP, FB, and SB) between two non-contact sports (i.e., BMT and VB). This study was used a 3D cube as stimulus in a mental body rotation task to assess cognitive performance. The aim was to examine if the dynamic balance would affect the performance of these athletes implying their use of motor processes during the task.

The results of our study indicate that there was a notable decrease in RT in both balance conditions (i.e., FB and SB) when compared to SP condition. This finding suggests that the unstable equilibrium position, experienced during the balance conditions, may have improved the cognitive processing abilities of the participants, enabling them to complete the MR task more quickly. In this line of idea, Kawasaki, Higuchi [47] demonstrated that MR interventions have immediate beneficial effects when used in dynamic balance conditions, and that the ability to mentally imagine the foot movement may be related to postural stability when involving a challenging postural task. Moreover, Kawasaki et al. [48] demonstrated that participants in unipedal standing performed the MR task faster than the quiet standing group and had lower sway scores. Also, it was discovered that the MR is involved in upright human posture control and may be related to the ability to stand as still as possible. It was noticed that MR performance was related to postural stability because both involve cognitive processes used for motor imagery as well as motor execution of the foot movement. Bigelow, Agrawal [20] confirm that vestibular function connects cognitive areas of visuospatial abilities (e.g., spatial memory, navigation, and mental rotation) and mental representation of three-dimensional space.

Furthermore, pairwise comparisons between MR conditions (i.e., SP, SB, and FB) revealed significant difference in RT in all degrees of rotation (i.e., 45°, 90°, 135°, 180°, 225°, 270°, and 315°) between SP vs. FB and between SP vs. SB conditions (*p* < 0.01) but we did not record significant result between FB vs. SB conditions. Specifically, at 135 degrees, players took longer time to respond to stimuli OC. In this context, Keehner et al. [49] and Michelon, Zacks [50] found that for egocentric MR tasks, RT tended to be longer at angles greater than 60° or 90°. Regardless of the increase in response time observed in our study, it is not linear with rotation angles, contrary to the findings of Shepard, Metzler [2], who demonstrated a linear increase in RT with increasing angular disparity between two presented stimuli. Therefore, we can argue that adding a second task (i.e., postural balance) may be responsible for increasing RT by diverting attention from the MR task. In fact, Huxhold et al. [51] and Shumway-Cook, Woollacott [52] both agree that postural stability is the result of shifting attention to cognitive tasks that increase the automation and efficiency of postural control processes. In addition, it should be noted that opposite angles (i.e., 45°-315°, 90°-270°, 135°-225°) converge RT values, possibly due to the right stimulus being a stimulus to the left mirror image. The same results were reported by Habacha et al. [15], Habacha et al. [39], Habacha et al. [16], and Steggemann et al. [1], but these authors used body rotation to confirm that MR tasks typically compute the mean RT of angular differences where the shortest rotational path between stimulus and target is the same. Wohlschläger, Wohlschläger [53] revealed that object-based transformations are the mental analogion to physical object manipulations in real space. Next to, the value of general EP did not show any significant difference between conditions (i.e., SP, FB, and SB) in all rotation degrees.

To test our second hypothesis, we compared the rotational ability of BMT and VB players. We found a significant group effect, showing that VB players take longer time to rotate objects mentally than BMT players at 45° (∆=23,47%) and 90° (∆=28,86%) angles (*p* < 0.05). This could be explained by the fact that in BMT the shuttlecock travels at a much higher speed and with a less predictable trajectory than in VB, so players need to have quick reflexes to be able to hit the shuttlecock accurately [54]. Many training drills in BMT require players to repeatedly produce the same responses to events. The BMT players may consequently have acquired the skill to quickly retrieve and encode information, resulting in enhanced response times, which may be transferable to visuo-spatial testing [27]. Even though both games are played across the net, BMT players must often respond fast to shots targeted directly at them, whereas VB players have more time to react because each player is aware of their location and function on the court. Furthermore, BMT players use information about their opponent facing the net to select what action to take [55] and receive a version of that information as if seeing in a mirror, necessitating a MR of these information before forecasting occurrences [27].

Our results revealed that the badminton players have better result on spatial imagery test and smaller error percentages than volleyball players. Shepard, Podgorny [56] linked MR and other image transformations to mechanisms underlying visual perception of movement. Furthermore, mental imagery cannot be understood without reference to space, which is inextricably linked to the concept of movement. indeed, the representation of space and the performance of quicker abilities entail a connection between the horizontal, vertical, and sagittal planes and sensorimotor system input [57]. We can take this explanation that BMT players’ physical manipulation of space and the use of a racquet for shuttle exchange may enhance their visuospatial concept grasp more than VB players who used bigger object during the game (i.e., ball) [58].

In addition, the analysis showed an interaction between group and balance appear in the general EP (*p* < 0.05). Let’s start with the idea that BMT and VB are two team sports, but the first is an individual sport (our case study), whereas VB is played with multiple players. In this case, we found that the BMT player’s perception only focuses on the opponent and the ball. This ability to locate opponents in the playing area is crucial and need to make quick decisions about where to move and what actions to take. This requires a good sense of spatial awareness, as well as the ability to mentally rotate objects to predict the trajectory of the shuttle or the movements of an opponent. However, in VB, the task of ball detection and tracking becomes more complex due to the presence of multiple players in a limited space. A high occlusion rate in ball images between players often means that direct detection methods fail [59]. Additionally, Carroll [22] explains to locate teammates and opponents in the playing area, the players move around the smaller on a smaller playing field while engaging in technical and tactical tasks. Thus, this requires a significant cognitive process, not only to understand one’s environment and move around in it, but also to perform a wide range of cognitive tasks that require the visualization of possible situational transitions.

The interaction between balance and groups, on the other hand, was significant in RT only at 225° angle in the SP vs. FB and SP vs. SB conditions (*p* < 0.05). Furthermore, the RT does not alter across FB and SB tasks. Stress/disturbance of postural balance, whether on the frontal or sagittal plane, causes the same reaction in both groups [60]. We continue by noting that this could be attributable to the motor task (i.e., balance), which appears to elicit nearly identical reactions in both fields (i.e., BMT and VB players) [61].

## Conclusion

In conclusion, this study confirmed the first hypothesis that dynamic balance conditions (i.e., FB and SB) on the wobble board SPBB have a direct beneficial effect on mental spatial capacities and MR of BMT and VB players, resulting in a decrease in their RT. According to the results, participants took longer to respond to object-cube stimuli at angles of 135°, 180°, and 225°, indicating the greatest difficulty in completing the MR task. Furthermore, BMT players had faster RT and less EP than VB players, despite representing the same progression kinetics of EP. Finally, dynamic balance is also an activity that involves mental manipulation of objects in 3D space, which can enhance BMT and VB players’ ability to rotate 3D cube stimuli.

## Data Availability

The datasets presented in this study can be available on demand at the first author.

## Abbreviations

BMT: Badminton
COM: Centre of mass
FB: Frontal balance
MR: Mental rotation
RT: Response times
SB: Sagittal balance
SPBB: Single plane balance board
SP: Standing position
VB: Volleyball
