# Correction: Does dynamic balance affect cube mental rotation task in badminton vs. volleyball female players?

**DOI:** 10.1186/s40359-024-01681-1

**Published:** 2024-04-11

**Authors:** Samiha Amara, Badriya Al-Hadabi, Heba El-Ashkar, Nabil Gmada, Hamdi Habacha, Bessem Mkaouer

**Affiliations:** 1 Physical Education and Sport Sciences Department, College of Education, Sultan Qaboos University, Muscat, Sultanate of Oman; 2 Higher Institute of Sport and Physical Education of Ksar Said, Manouba University, Tunis, Tunisia

**Correction to: BMC Psychology (2024) 12:131**

10.1186/s40359-024-01589-w

Following publication of the original article, it came to the attention of the journal that affiliation ‘2’ was incorrect because a correction to the affiliation was not implemented during proofing of the article. The affiliation has since been corrected in the published article. The publisher thanks you for reading this erratum and apologizes for any inconvenience caused.

